# Clinical Transition Experiences of Internationally Educated Nurses Into Major Trauma Care: A Descriptive Phenomenological Study

**DOI:** 10.1155/jonm/8827306

**Published:** 2026-05-25

**Authors:** Adam Ash, Kay De Vries, Jane Rutty

**Affiliations:** ^1^ School of Nursing and Midwifery, University of Birmingham, Birmingham, England, UK, birmingham.ac.uk; ^2^ School of Nursing and Midwifery, De Montfort University, Leicester, England, UK, dmu.ac.uk; ^3^ East Midlands Forensic Pathology Unit, University of Leicester, Leicester, England, UK, le.ac.uk

**Keywords:** clinical transition, descriptive phenomenology, emotional labour, internationally educated nurses, trauma nursing, workforce integration

## Abstract

**Aim:**

To explore the lived clinical transition experiences of internationally educated nurses entering major trauma care without prior trauma nursing experience.

**Design:**

Descriptive phenomenological study.

**Methods:**

Unstructured in‐depth interviews were conducted twice with nine internationally educated nurses (seven female; mean age 32.5 years) working in a Level I major trauma centre in Saudi Arabia between February and April 2021. All participants had prior clinical experience but no trauma background. Colaizzi’s seven‐step method guided analysis, supported by field notes and member checking. Husserl’s phenomenological attitude was maintained to ensure bracketing of preconceptions.

**Results:**

Three interrelated themes described transition: facing a new reality, adjusting towards growth and achieving belonging. Despite established nursing backgrounds, participants reverted to novice status in trauma care, marked by fear, uncertainty, and procedural unfamiliarity. Repeated exposure, mentorship and team inclusion gradually enhanced competence and confidence. Emotional labour was central, enabling nurses to suppress fear, maintain composure and progressively reconstruct resilience and professional identity in high‐acuity practice.

**Conclusion:**

The transition of internationally educated nurses into trauma care involves navigating high‐stakes demands as novices, with emotional labour functioning as both a protective and developmental mechanism.

**Implications for Nursing Management:**

Structured trauma‐specific transition pathways, including simulation and mentorship, are needed to support internationally educated or novice nurses entering high‐acuity services. Organisational recognition of the emotional demands of clinical transition can strengthen integration, retention and workforce sustainability in trauma systems.

**Impact:**

This study addressed the challenge of how internationally educated nurses adapt to major trauma care without prior experience. It found that transition required returning to novice status and relying on emotional labour to build resilience and professional identity. These findings have global relevance for workforce integration in trauma or high‐acuity care systems reliant on internationally educated staff.

**Reporting Method:**

The study adhered to COREQ guidelines.

**Patient or Public Contribution:**

No patient or public contribution.

**What This Paper Contributes to the Wider Global Clinical Community**
• Provides the first in‐depth phenomenological account of how internationally educated nurses clinically transition into high‐acuity trauma care, showing that even experienced practitioners often revert to novice status when entering advanced trauma systems.
• Demonstrates how emotional labour functions as a protective mechanism against stress and also as a developmental strategy, enabling resilience, competence and identity reconstruction in trauma nursing.
• Highlights the need for trauma‐specific transition and onboarding programmes, including simulation, mentorship and early team integration, to accelerate readiness and retention of internationally educated nurses in high‐demand global trauma systems.

## 1. Introduction and Background

The migration of nurses across borders has accelerated in recent decades, driven by persistent workforce shortages, ageing populations, and growing healthcare demands [1]. Consequently, many high‐income countries now rely structurally on internationally educated nurses to sustain safe staffing levels [2, 3]. This reliance has been reinforced by the COVID‐19 pandemic, which exposed vulnerabilities in national health workforces and increased the need for international recruitment ([4]; NSI, 2023).

Internationally educated nurses contribute valuable expertise, but successful integration depends on safe and effective transition into host healthcare systems. Reported challenges include differences in scope of practice, clinical protocols, interprofessional communication and the availability of structured orientation [5–7]. Transition programmes often emphasise cultural or organisational adaptation while overlooking the speciality‐specific competencies required in high‐acuity settings [8, 9]. Inadequate preparation leaves internationally educated nurses underequipped for the technical, cognitive and emotional demands of specialty practice, with implications for patient safety, staff wellbeing and workforce retention [10].

The lack of research on speciality‐focused transitions is well‐recognised. Most studies on internationally educated nurses concentrate on sociocultural adaptation, communication and discrimination, with limited examination of role integration and clinical demands in specialties such as critical care, emergency and trauma [11–14]. Addressing this gap is essential to design interventions that prepare internationally educated nurses for high‐acuity roles with confidence and competence.

Major trauma care is one such specialty where inadequate preparation can have serious consequences [15–17]. Trauma is a leading cause of preventable mortality and long‐term disability, accounting for millions of deaths each year [18, 19]. Organised trauma systems improve outcomes through coordinated multidisciplinary care, rapid triage and timely interventions [19, 20]. Nurses are central to these systems, providing initial resuscitation, haemorrhage control, airway and ventilation support, massive transfusion activation, advanced monitoring and interprofessional coordination [21]. These responsibilities require advanced technical expertise, situational awareness and the ability to perform under extreme time pressure [22, 23].

Transition into trauma care is challenging for all nurses, but internationally educated nurses without prior trauma background experience the added burden of adapting simultaneously to a new healthcare system and country. This process involves learning unfamiliar clinical protocols, operating advanced technologies, adjusting to diverse communication styles and integrating within established multidisciplinary teams [24, 25]. Trauma nursing was selected as the focus because it represents one of the most complex and high‐acuity areas of practice, requiring rapid clinical judgement, interdisciplinary coordination and sustained exposure to critically injured patients (Glen et al., 2016; [27]). Despite global reliance on internationally recruited nurses, limited evidence explains how they adapt clinically within these demanding environments. Inadequate specialty‐specific preparation has been shown to hinder competence development, erode confidence and heighten stress [5–7], all of which can affect team cohesion and care quality in trauma systems [25].

## 2. The Study

Given the growing reliance on internationally educated nurses, the complexity of trauma care, and the need to build sustainable trauma nursing workforces, it is essential to understand how internationally educated nurses experience clinical transition into this speciality. Evidence from such research can inform targeted onboarding, mentorship and continuing professional development strategies that reflect the realities of trauma practice. This study, therefore, explores the lived clinical transition experiences of internationally educated nurses entering major trauma care, addressing a critical gap in the literature.

## 3. Methods

### 3.1. Design

A descriptive phenomenological approach grounded in Edmund Husserl’s (1859–1938) philosophy was used to explore the lived clinical transition experiences of internationally educated nurses entering major trauma care without prior trauma nursing experience. Descriptive phenomenology seeks to capture how phenomena are experienced by individuals and aims to identify their essential structures and associated meanings [28]. Husserl emphasised that experience is the foundation of meaningful knowledge and that revealing the essence of phenomena requires researchers to adopt a phenomenological attitude by setting aside preconceptions through *epoché* and reduction [28]. This study applied Husserl’s principles of intentionality and phenomenological reduction, ensuring that the phenomenon was approached with openness and that findings reflected participants’ own accounts rather than researcher interpretations. Descriptive phenomenology was chosen over interpretive phenomenology, as the researchers did not share the participants’ experiences and therefore sought to remain close to participants’ descriptions without imposing additional interpretation [30]. The study adhered to COREQ guidelines as presented in Supporting Material B.

### 3.2. Study Setting and Recruitment

The study was conducted in a Level I major trauma centre within a large tertiary referral hospital in Saudi Arabia. The centre holds the highest national trauma designation but does not have formal verification from the American College of Surgeons (ACS) [19, 31, 32]. The centre’s high trauma caseload, comprehensive range of services and substantial proportion of internationally educated nurses made it an appropriate and information‐rich setting for exploring clinical transition from nontrauma backgrounds.

Level I trauma centres deliver the highest level of specialist trauma care, providing 24‐h access to multidisciplinary teams, advanced surgical and critical care services and integrated trauma systems aligned with international benchmarks [20]. These characteristics ensured that participants worked within a high‐acuity environment representative of global trauma care systems.

Participants were recruited between February and April 2021 through flyers and response slips distributed in clinical areas, using purposive sampling to select individuals most able to provide rich and relevant accounts of the phenomenon [33]. Purposive sampling is widely used in phenomenological research because it prioritises depth over breadth and ensures inclusion of participants with direct experience of the phenomenon under study [34]. Participation was voluntary, with no financial incentives or honoraria offered. No prior relationship existed between researchers and participants.

### 3.3. Inclusion and Exclusion Criteria

Inclusion criteria were (1) nurses trained outside the host country, (2) at least 1 year of current experience in the trauma centre, and (3) no prior trauma nursing experience in their home country or elsewhere. Nurses with previous trauma nursing experience, those employed in temporary positions, and locally trained nurses were excluded. Specifically, locally trained nurses at the same institution typically undertake a longer preceptorship period but do not receive formal trauma‐specific training. They usually spend an extended period in the general emergency department before being assigned to the trauma centre; therefore, they were excluded from participation. Consequently, a total of 20 nurses expressed interest and returned signed response slips. Subsequently, participant information sheets were sent to them detailing all aspects of participation, and nine ultimately participated in the study.

### 3.4. Data Collection

Data were collected by the main researcher through unstructured, individual in‐depth interviews conducted in English in a quiet, private room within the emergency and trauma department. Interviews lasted from 60 to 90 min. In keeping with descriptive phenomenology, interviews were unstructured to enable participants to articulate their experiences in their own words [35, 36]. Each interview began with the broad question: “What is your experience in moving to major trauma care?” Probing questions explored specific clinical tasks, challenges, learning strategies and team integration, with most probes arising naturally during the conversation.

Throughout data collection, the researcher maintained a phenomenological attitude by bracketing personal assumptions and remaining open to participants’ lived experiences [28, 28, 37]. Each participant took part in two interviews. The second interview enabled member checking and clarification, with participants provided with their first interview transcript to review. They were invited to remove or amend any content and to elaborate on earlier responses. Field notes captured contextual observations and reflexive insights, supporting both analytical depth and methodological rigour [38, 39].

Conventional data saturation was not the primary objective in sampling and data collection for this study. In phenomenological research, the emphasis is placed on the depth and richness of each participant’s experience. The goal is to understand the essence of the phenomenon rather than to simply reach a point where no new insights are produced [39–41]. Therefore, the study aimed to recruit a small sample of participants and to evaluate adequacy based on the richness of the narratives. After the ninth participant, data collection was concluded.

### 3.5. Data Analysis

All interviews were audio‐recorded, anonymised, and transcribed verbatim. Data were analysed using Colaizzi’s [42] seven‐step method, which was selected for its congruence with descriptive phenomenology and its requirement for participant validation of findings [43]. Colaizzi’s method was chosen over other descriptive phenomenological approaches, such as those of Giorgi or Van Kaam, because it offers a structured and transparent procedure while explicitly incorporating participant validation, thereby enhancing credibility and trustworthiness [28, 44].

The analytic process involved: (1) reading all transcripts to acquire a sense of the whole, (2) extracting significant statements, (3) formulating meanings, (4) organising formulated meanings into theme clusters, (5) developing an exhaustive description, (6) identifying the fundamental structure of the phenomenon, and (7) returning to participants for validation. NVivo v12 software supported data management and coding. Data were coded by the main author, in which no themes were identified before the coding process, and subsequently verified by the research team. A detailed step‐by‐step account of the application of Colaizzi’s method is provided in Supporting Material A.

The phenomenological attitude was sustained throughout the analysis by ongoing reduction and bracketing. The main researcher documented preconceptions and prior assumptions in a reflexive diary before and during analysis, revisiting these notes at each stage to ensure they did not influence coding or theme development. Transcripts were read multiple times with deliberate suspension of presuppositions, focusing only on the intentional relationship between participants and the phenomenon [28, 28, 37]. Regular peer debriefing with the research team provided an additional layer of reflexive questioning, further supporting phenomenological reduction. These steps ensured the analysis remained grounded in participants’ accounts rather than researcher interpretation [45].

### 3.6. Ethical Considerations

The study was conducted in accordance with the ethical principles of the Declaration of Helsinki [46]. Ethical approval was obtained from the local Institutional Review Board (Ref. H1RI‐30‐Jan19‐02) and the De Montfort University Faculty of Health and Life Sciences Research Ethics Committee (Ref. 3175). Written informed consent was secured from all participants after they had received both verbal and written information about the study. Confidentiality was safeguarded by using pseudonyms, secure data storage, and anonymisation of transcripts. Participants were reminded of their right to withdraw from the study at any stage without consequence.

### 3.7. Rigour and Reflexivity

The phenomenological attitude requires a deliberate shift from the natural attitude, which accepts the world as it appears, to a reflective stance aimed at uncovering the essence of experience [28]. This involves *epoché*, or bracketing, whereby pre‐existing knowledge, beliefs, and assumptions are suspended so that the phenomenon can be understood as described by participants [30, 37]. In this study, bracketing was supported through reflexive journaling and regular discussions among the research team, who brought diverse clinical backgrounds and perspectives. These strategies ensured that the study remained aligned with the core principles of descriptive phenomenology [30].

Trustworthiness was established using Lincoln and Guba’s [47] criteria, supported by more recent methodological guidance on ensuring rigour in qualitative health research [48, 49]. Credibility was enhanced through prolonged engagement, two interviews per participant, member checking, and peer debriefing with a qualitative research expert external to the study. Transferability was supported by providing thick descriptions of the research context, participants, and procedures. Dependability was maintained through a transparent decision trail documenting all methodological and analytical steps. Confirmability was ensured through an audit trail and reflexive diary, safeguarding that findings reflected participants’ lived experiences rather than researcher bias.

## 4. Findings

This paper presents the clinical transition experiences of a group of internationally educated nurses entering major trauma care without prior trauma nursing experience. Broader cultural and social adaptation themes, also identified in the full dataset, are reported separately.

### 4.1. Characteristics of Participants

Nine nurses participated: seven women and two men, with a mean age of 32.5 years (range 25–39). Five participants came from the Philippines, and four were from India. All held a Bachelor of Science in Nursing and had previously worked in nontrauma areas such as medical, surgical, or emergency wards, with an average of 3.9 years of experience in their home countries. None had prior trauma nursing experience. At the time of the interviews, participants had an average of 6.2 years of local trauma experience (range 2–12). Two participants had more than 10 years of trauma experience; however, their accounts were retained, as their transition experiences reflected similar processes to the rest of the cohort.

Upon employment, all participants completed a brief hospital‐wide orientation lasting 1 to 2 weeks but received no structured trauma‐specific onboarding or simulation training. They were allocated directly to trauma units following a short period of shadowing and initial shifts in the general emergency department.

The clinical transition journey was structured into three main themes and associated subthemes, summarised in Table 1. These themes capture the sequential and overlapping nature of the participants’ journey from facing a new reality to professional growth and eventual belonging.

**TABLE 1:** Thematic structure of the clinical transition journey of internationally educated nurses.

Main theme	Subthemes
Facing a new reality	• Entering an unfamiliar trauma hotspot
	• Dealing with complexity and unpredictability

Adjusting towards growth	• Building competence through exposure
	• Transforming perspectives and professional identity

Achieving belonging	• Being in control
	• Expressing strength, confidence and pride

*Note:* Thematic structure of the clinical transition journey of internationally educated nurses into major trauma care. Three main themes and their subthemes were identified through descriptive phenomenological analysis, reflecting the phased trajectory from initial disorientation to eventual belonging.

### 4.2. Facing a New Reality

The first stage of transition was characterised by participants’ immersion into a trauma care environment that felt profoundly unfamiliar. Although they entered with established nursing experience, they encountered situations that rendered their prior knowledge inadequate. This sudden exposure to high‐acuity trauma practice disrupted their sense of competence and security, confronting them with both clinical and emotional challenges that defined the beginning of their transition journey.

Participants described anxiety about being assigned to critical cases without trauma‐specific preparation, though open resentment was rarely expressed. The absence of structured support or transition programmes further intensified these early challenges, leaving nurses to navigate their new roles largely through trial and self‐reliance.

#### 4.2.1. Entering an Unfamiliar Trauma Hotspot

Participants described their initial experiences as an intense clinical shock. On their first shifts, they were met with crowded trauma bays, filled with simultaneous activity and multiple professionals working under intense time pressure. They were immediately expected to contribute, while trying to make sense of the speed, the equipment, and the severity of patient injuries.

“…this open fracture was the first time for me to see it in my life. It was a special case”. (P07)

“Patients here are more complex… I didn’t handle trauma in the Philippines, but I handle all kinds of trauma here. Most cases are road traffic accidents.” (P06)

These encounters destabilised their confidence, as the familiar logic of ward‐based care did not apply in trauma bays where events unfolded in minutes, and silence or hesitation could have direct consequences for patients. The sudden immersion generated feelings of being overwhelmed and exposed, as though they were constantly “*catching up*” with the pace of care.

The trauma environment was described as volatile and unforgiving. Periods of relative calm were disrupted without warning by the simultaneous arrival of several critically injured patients. Participants spoke of this unpredictability as both a physical and psychological burden, leaving little space to recover or prepare for what came next.

“It’s unpredictable… within one hour all patients will get in together. Sometimes there’s no break even to use the comfort room [rest room]”. (P01)

The pressure of time, the noise, and the unfamiliar technology compounded their sense of inadequacy. They recalled struggling to complete tasks within expected timeframes, fearing that hesitation would be visible to their colleagues and detrimental to their patients.

“Initially… I wasn’t sure if I’m doing my job the right way.” (P03)

“There were challenges with abdominal trauma cases and polytrauma. At first it was a struggle… dealing with multiple or polytrauma cases, it was really difficult to know how to transfer the patient for example” (P04)

The essence of this experience was one of being abruptly displaced from the comfort of the known into a space that demanded speed, precision, and calm under pressure. Their entry into trauma care was therefore experienced as disorientation, a stripping away of prior competence, and a demand to relearn themselves as nurses within an environment mostly described as unfamiliar.

#### 4.2.2. Dealing With Complexity and Unpredictability

The intensity of trauma care was technical and deeply emotional. Participants described the shock of seeing patients who appeared stable collapse without warning, leaving them to encounter the immediacy of life and death in ways they had never experienced before.

“A patient came after an RTC [Road Traffic Collision), talking to me… then suddenly he got arrested. Just in the span of two hours, he died”. (P06)

“Initially he looked like a simple case… then he became hypotensive and deteriorated suddenly. We need to be prepared”. (P05)

Such experiences ignited self‐doubt and guilt, as participants questioned whether they had acted quickly enough or whether different actions might have changed the outcome. In these early stages, responsibility was internalised, and death was felt as a personal failure rather than a reflection of the unpredictable nature of trauma.

“At the beginning, I was blaming myself, like. Did I not do enough, so they died?” (P06)

Caring for patients who mirrored their own age or family circumstances intensified this impact. Encounters with young adults or children triggered strong feelings of sadness and guilt, not only for the patients themselves but also through imagining similar injuries happening to their own families. Being abroad, away from their loved ones, heightened this distress, as participants worried what might happen if such events occurred while they were not present.

“Sometimes I felt very guilty because patients were the same age as me… sometimes paralysed… I felt like it was happening to my own family, and if I carried that guilt all the time, I would not be able to work here as a nurse” (P02)

“I feel sad for them [children with trauma], why this happened to this child”. (P08)

While the emotional weight was heavy, participants also described the necessity of concealment. They had to present a composed exterior even when internally shaken, maintaining the performance of calmness in front of colleagues and patients.

“Even if I was panicking inside, I had to look fine in front of everyone” (P03).

Others echoed this, describing how emotions were displaced into private spaces, hidden from view until the end of a shift.

“I don’t cry at work; I cry at home, alone… people know me as always smiling, but at the end of the day I feel exhausted” (P06).

The essence of this phase was therefore characterised by a dual struggle: the technical challenge of acting competently in an unfamiliar clinical environment and the emotional labour of containing distress to continue functioning within it.

### 4.3. Adjusting Towards Growth

The second stage of transition reflected a gradual adjustment, where the initial turbulence of entry gave way to adaptation and professional growth. Through sustained exposure to high‐acuity trauma, participants described a process of building competence, reframing challenges and redefining themselves as trauma nurses. Fear and uncertainty progressively shifted into confidence, emotional resilience and a stronger sense of professional purpose.

#### 4.3.1. Building Competence Through Exposure

Participants emphasised that repeated exposure to trauma was critical to their growth. With each case, what had once provoked fear gradually became more familiar. The shock of managing open fractures, polytrauma or sudden deterioration gave way to a rhythm where recognition, anticipation and action felt increasingly instinctive. They described being forced to act before they felt ready, then realising afterwards that they had grown more capable as a result:

“…the more you see, the more you will know. We became stronger by seeing more cases and understand real facts about what we do”. (P05)

“When I started my work as a trauma nurse, I was afraid… but by seeing these cases regularly, I feel that I’m used to them. Now I can handle any type of patient.” (P02)

Technical skills such as inserting chest drains, performing complex transfers or assisting in emergency thoracotomy were not acquired through gradual classroom learning but through direct, high‐stakes encounters. This hands‐on practice embedded competence in ways that no amount of preparation could have replicated.

“Body wise, it [being a trauma nurse] makes me like tired and stressed, but knowledge wise, it makes me more effective”. (P06)

At this stage, participants also internalised a deeper ethic of prioritisation. The patient became the absolute focus, with personal needs temporarily suspended in the intensity of care.

“It’s in our line of work that we keep the patient first… they need urgent care, like for example that patient needs a chest tube or a Foley’s catheter or a simple dressing… sometimes it’s okay for us, or for me not to take a break or go to the restroom”. (P09)

The unpredictability that once paralysed them was now interpreted differently. Instead of being destabilising, it became stimulating, a form of challenge that demanded alertness and competence.

“It’s exciting now… in trauma you don’t know what’s happening next, and it’s always a surprise. That’s what I love… every day, new cases, new patients.” (P03)

The essence of this subtheme lies in transformation through repeated exposure. What was once frightening and disorienting became the foundation of competence and resilience. Growth was forged through immersion, where exhaustion coexisted with mastery, and uncertainty evolved into motivation.

#### 4.3.2. Transforming Perspectives and Professional Identity

Growth extended beyond technical ability into how participants understood themselves as nurses and as people. They spoke of “*becoming stronger*” by mastering procedures, regulating emotions, prioritising under pressure, and accepting that not all outcomes could be controlled. Death, which at first had been experienced as personal failure, gradually came to be understood as an inevitable reality of trauma care.

“Before, I used to feel sad when a patient died. Then, as time passed, I had the feeling that I tried my best, and this is what I could do. The experience changed me a lot”. (P06)

Part of this transformation involved a new relationship with time. Where early encounters left them feeling rushed or disoriented, they now described anticipating deterioration, intervening earlier and aligning their actions with the rhythm of the trauma team.

“At first, I was so nervous, but when I came to encounter trauma patients every day, I know the sequences of treatment, so I’m anticipating those things.” (P03)

The essence of this phase was adjustment and redefinition. The transition shifted from merely surviving trauma care to re‐imagining nursing itself. Participants built emotional resilience, reframed unpredictability as opportunity, and developed confidence in their ability to act effectively in complex, uncertain situations.

### 4.4. Achieving Belonging

The final stage of transition was marked by a sense of belonging and mastery in trauma nursing. What had once felt unfamiliar and overwhelming was now experienced as routine, manageable and even a source of pride. Nurses spoke of advanced expertise, confidence and strength, a culmination of their growth across previous phases.

#### 4.4.1. Being in Control

By this stage, participants described a capacity to manage trauma care with assurance and composure. Years of accumulated exposure had transformed urgent situations that once felt chaotic into structured routines. They were able to anticipate sequences of care, prioritise under pressure and sustain performance even during prolonged busy shifts:

“…even if it’s busy, we can manage it because this is what we have been dealing with as a routine”. (P04)

“By working here, I know I must have a long patience. I knew how to handle stressful situations and how to handle bad situations”. (P05)

Control was technical and emotional. Where early experiences of patient death had triggered sadness and guilt, later encounters were managed with acceptance and perspective:

“Before, when I lost a patient, it was very sad. But now I can adapt. Everybody tries their best, but sometimes we still lose them. I accept that maybe this patient cannot live anymore”. (P08)

This emotional regulation extended to interactions with patients and families. Rather than reacting with frustration, they described calming tense situations and containing the atmosphere around the patient:

“When the family gets in rushing, someone must be calm, so you stay focused on what you’re doing. If you panic, nothing will be done.” (P01)

Control thus reflected professional maturity, the ability to manage the technical pace of trauma care and the emotions surrounding it. Nurses saw themselves not just as task executors but as steadying figures within the wider team and environment.

#### 4.4.2. Expressing Strength, Confidence and Pride

Alongside control, participants articulated a deep sense of strength, confidence and pride. The same work that had once left them in tears was now a source of fulfilment and empowerment.

“When I started my career here, I was sensitive. Sometimes we were crying, but with this experience I feel stronger. I’m not crying anymore.” (P05)

Many described this strength in terms of professional identity, by being competent and embodying the role of a trauma nurse with authority and assurance.

“Now I feel that I’m a certified emergency nurse. I’m so proud of myself that I can work anywhere… ICU, CCU, trauma. I can be there. I can give you the satisfaction you want.” (P01)

“Being a trauma nurse, not only in the hospital. If any disaster happened and I am present, I can manage. I am confident that I can manage anywhere”. (P02)

Pride was grounded in reflection on how far they had come, from initial fear and self‐doubt to becoming role models for others.

“When I came here, I knew only a few things. Now I know how to handle patients systematically. And I can educate junior staff on how to deal with trauma patients”. (P02)

Growth was also marked by recognition of belonging within the team. Being involved in complex, high‐stakes procedures symbolised their transformation from newcomers to integral members:

“Now, we have a fully equipped trauma team, and I feel I am part of it… by the experience, you will change. You will not be the one when you started your career”. (P05)

At this stage, nurses contrasted their early uncertainty with their current confidence and direction:

“I think today is different because I’m not like before when I came here, I’m much more confident. I had only a little experience about trauma patients when I first came here.” (P08)

“…here every day is different. That’s why I don’t want to go back to the ward.” (P09)

The essence of this stage was not simply proficiency but belonging. Nurses no longer spoke of “catching up” or “surviving.” Instead, they voiced pride in mastery, resilience in adversity, and confidence in their ability to contribute meaningfully to complex, life‐saving care.

## 5. Discussion

This study explored the clinical transition of internationally educated nurses into major trauma care through longitudinal recall of lived experiences. Whereas much of the internationally educated nurses’ literature has emphasised sociocultural adaptation and system‐level challenges, including communication norms and organisational differences [1, 14, 50, 51], our findings show that the clinical dimension of transition is equally critical. Participants’ accounts demonstrated that emotional labour was the central mechanism through which they navigated trauma nursing, enabling them to withstand immediate pressures, regulate vulnerability and progressively build resilience and professional identity. This extends the evidence base by highlighting clinical transition in internationally educated nurses and by situating emotional labour within a specialist nursing context where the stakes are acute and life‐threatening rather than service oriented.

Early transition was marked by disorientation, fear, and lack of confidence, consistent with research on internationally educated nurses documenting uncertainty and stress in initial phases [52, 53]. The trauma setting intensified these challenges through constant exposure to death, severe injuries and rapid deterioration. Such conditions reflect the intrinsic pressures of emergency and trauma nursing, where unpredictability, immediacy, and high‐stakes decision‐making are defining features and where stress and burnout are well‐recognised risks [54–57]. Yet, the same repeated exposure that produced exhaustion and emotional strain also became the foundation of growth. Situational learning in high‐acuity encounters enabled nurses to transform fear into anticipation, uncertainty into confidence and vulnerability into resilience. These findings reinforce the importance of experiential learning to professional development [58, 59], while drawing attention to the personal cost attached to this process.

The trajectory of development aligned closely with Benner’s [60] Novice to Expert framework. Despite years of prior nursing experience, participants entered trauma care as novices, relying on rules and struggling with uncertainty. Over time, repeated immersion in complex cases facilitated progression towards competence and proficiency, reflected in improved anticipation, pattern recognition, and integration into the trauma team. While few described the intuitive grasp associated with expert practice, their narratives showed steady movement along Benner’s continuum [61]. This reinforces the premise that expertise is context‐specific and that experienced practitioners may revert to novice status when entering unfamiliar specialties [62, 63]. Importantly, however, skill acquisition alone cannot fully explain the process of adaptation observed. Emotional labour underpinned the ability to sustain this developmental trajectory under extreme conditions.

Transitioning to trauma care as novice internationally educated nurses was experienced as both demanding and transformative. While structured transition programmes have been shown to buffer early challenges [64], their absence in this setting meant participants relied almost entirely on emotional labour. Nurses spoke of suppressing fear, concealing distress, and projecting composure in high‐pressure encounters. In line with Hochschild’s [65] concepts of surface and deep acting, emotional labour functioned both as a protective mechanism and as a catalyst for growth. It enabled sustained performance under pressure, promoted belonging within the trauma team and supported the reframing of unpredictability as purposeful challenge, thereby enhancing resilience across the transition journey [66]. This interpretation challenges the dominant view of emotional labour as primarily detrimental, often linked with burnout and compassion fatigue [67, 68], by illustrating its constructive role in adaptation and identity reconstruction. Emergency nurses are bound by feeling rules that require them to appear composed even when distressed [69]. As this study demonstrates, emotional labour extended beyond concealment, becoming a mechanism through which resilience was actively cultivated and professional identity redefined. In this sense, emotional labour was not simply an additional burden but a resource that enabled internationally educated nurses to move from survival to confidence and belonging in high‐acuity care.

### 5.1. Strengths and Limitations

This study has several strengths. It applied a descriptive phenomenological approach, supported by Colaizzi’s method, ensuring systematic and transparent analysis grounded in participants’ lived accounts. Rigour was enhanced through the use of bracketing, reflexive journaling, and peer debriefing, helping to minimise researcher bias. Conducting two interviews with each participant and incorporating member checking added further credibility and depth, while the focus on a Level I trauma centre provided a rich context for exploring transition in a high‐acuity setting rarely examined in existing literature.

There are also some limitations. Data were generated through interviews and field notes only, which may have restricted opportunities to capture additional perspectives on the phenomenon. Complementary approaches such as participant observation, document analysis or perspectives from managers and colleagues might have provided further contextual insights. Additionally, interviews were conducted in English, which was not the participants’ first language; this may have constrained the articulation of subtle meanings, despite clarification opportunities and member checking. Finally, the researchers’ nontrauma background could have shaped the focus of interviews and interpretation, though methodological safeguards supported adherence to phenomenological principles and helped mitigate this influence.

### 5.2. Implications for Practice, Policy and Future Research

Given the ongoing global reliance on internationally educated nurses, it is inevitable that they will continue to be allocated across diverse clinical areas, including highly specialised settings such as trauma. This reality highlights the need for greater attention to their transition journeys. Clinical practice should prioritise structured transition support, including mentorship and simulation‐based orientation, to equip internationally educated nurses with the skills and emotional resources required for high‐acuity care. At a policy level, workforce planning should recognise the dual clinical and emotional demands placed on internationally educated nurses and ensure that support frameworks extend beyond technical training to include resilience‐building and integration strategies. Future research should examine how different models of transition support influence internationally educated nurses’ retention, performance and patient outcomes in specialist settings. Comparative studies across clinical contexts would be particularly valuable in determining which approaches are most effective and sustainable.

## 6. Conclusion

This study has shown that the clinical transition of internationally educated nurses into major trauma care is shaped by intense demands and sustained through the practice of emotional labour. Entering trauma care reset experienced nurses to a novice state, but repeated immersion in high‐acuity encounters enabled growth towards competence and resilience. Emotional labour was not only protective but also developmental, allowing nurses to regulate vulnerability, integrate into the trauma team and reconstruct professional identity. By positioning emotional labour as a central mechanism of clinical transition, this study extends current understandings of internationally educated nurses’ adaptation and highlights the need for structured support that acknowledges both the technical and emotional dimensions of trauma nursing.

## Author Contributions

Adam Ash: conceptualisation, methodology, project administration, data curation, formal analysis, software, writing–original draft, and writing–review and editing. Kay de Vries: methodology, supervision, and validation. Jane Rutty: methodology, supervision, and validation.

## Funding

This research did not receive any specific grant from funding agencies in the public, commercial, or not‐for‐profit sectors.

## Conflicts of Interest

The authors declare no conflicts of interest.

## Supporting Information

Additional supporting information can be found online in the Supporting Information section.

## Supporting information


**Supporting Information 1** Supporting Material A provides a detailed step‐by‐step account of how Colaizzi’s seven‐step method was applied in this study.

**Supporting Information 2** Supporting Material B presents the Consolidated Criteria for Reporting Qualitative Research (COREQ) checklist.

## Data Availability

The data included in this published article are available upon request to the corresponding author.
