# Supplementary material for: Clinical Transition Experiences of Internationally Educated Nurses Into Major Trauma Care: A Descriptive Phenomenological Study
Source: J Nurs Manag. 2026 May 25;2026:8827306. doi: 10.1155/jonm/8827306 (PMC13201905; doi:10.1155/jonm/8827306)
Supplement: Supplementary file 1 — Supporting Information 1 Supporting Material A provides a detailed step‐by‐step account of how Colaizzi’s seven‐step method was applied in this study. [file JONM-2026-8827306-s001.pdf]

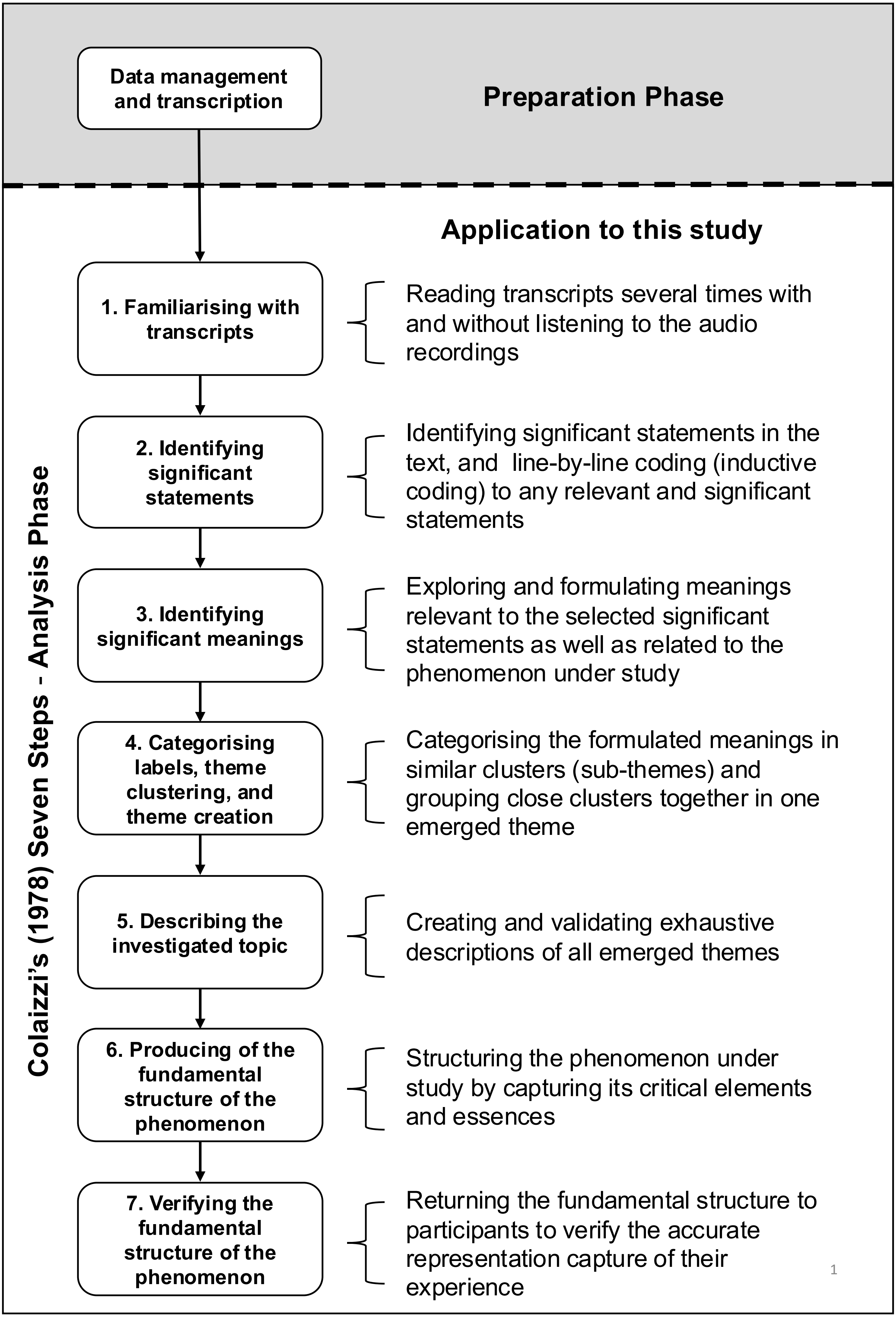
Data management and transcription
Preparation Phase
Application to this study
Colaizzi's (1978) Seven Steps - Analysis Phase
1. Familiarising with transcripts
Reading transcripts several times with and without listening to the audio recordings
2. Identifying significant statements
Identifying significant statements in the text, and line-by-line coding (inductive coding) to any relevant and significant statements
3. Identifying significant meanings
Exploring and formulating meanings relevant to the selected significant statements as well as related to the phenomenon under study
4. Categorising labels, theme clustering, and theme creation
Categorising the formulated meanings in similar clusters (sub-themes) and grouping close clusters together in one emerged theme
5. Describing the investigated topic
Creating and validating exhaustive descriptions of all emerged themes
6. Producing of the fundamental structure of the phenomenon
Structuring the phenomenon under study by capturing its critical elements and essences
7. Verifying the fundamental structure of the phenomenon
Returning the fundamental structure to participants to verify the accurate representation capture of their experience

Supplementary Material A

Step-by-step application of Colaizzi's (1978) seven-step method in this study. The process included preparation and analysis phases: familiarisation with transcripts, extraction of significant statements, formulation of meanings, clustering into sub-themes and themes, development of exhaustive descriptions, identification of the fundamental structure, and participant validation.
